# (*Z*)-Ethyl 2,4-diphenyl-3-(propyl­amino)­but-2-enoate

**DOI:** 10.1107/S160053680804395X

**Published:** 2009-01-08

**Authors:** Huimin Jin, Peifan Li, Bin Liu, Xiaoqing Cheng

**Affiliations:** a Department of Pharmaceuticals, Tianjin Medical College, Tianjin 300222, People’s Republic of China

## Abstract

The title compound, C_21_H_25_NO_2_, adopts a *Z* conformation about the C=C double bond. The mol­ecular structure is stabilized by an intra­molecular N—H⋯O hydrogen bond and the dihedral angle between the aromatic ring planes is 76.04 (12)°. The atoms of the ethyl substituent are disordered over two sets of sites in a 0.60 (2):0.40 (2) ratio.

## Related literature

For the synthesis, see: Du *et al.* (2006 ▶). For background, see: Xue *et al.* (2007 ▶).
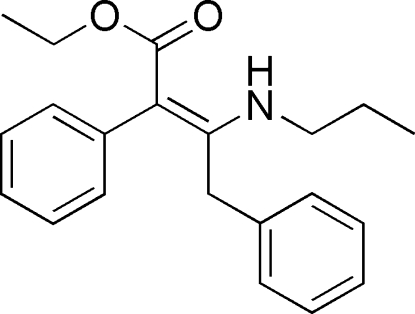


## Experimental

### 

#### Crystal data

C_21_H_25_NO_2_
*M* *_r_* = 323.42
Monoclinic, 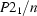

*a* = 12.186 (2) Å
*b* = 8.4771 (17) Å
*c* = 19.080 (4) Å
β = 106.33 (3)°
*V* = 1891.4 (7) Å^3^
*Z* = 4
Mo *K*α radiation
μ = 0.07 mm^−1^
*T* = 293 (2) K
0.28 × 0.22 × 0.18 mm

#### Data collection

Rigaku Saturn diffractometer
Absorption correction: multi-scan (*CrystalClear*; Rigaku/MSC, 2005 ▶) *T*_min_ = 0.980, *T*_max_ = 0.987
12325 measured reflections
3323 independent reflections
2290 reflections with *I* > 2σ(*I*)
*R*_int_ = 0.034

#### Refinement

*R*[*F* ^2^ > 2σ(*F* ^2^)] = 0.060
*wR*(*F* ^2^) = 0.198
*S* = 1.06
3323 reflections
232 parameters
5 restraints
H atoms treated by a mixture of independent and constrained refinement
Δρ_max_ = 0.24 e Å^−3^
Δρ_min_ = −0.19 e Å^−3^

### 

Data collection: *CrystalClear* (Rigaku/MSC, 2005 ▶); cell refinement: *CrystalClear*; data reduction: *CrystalClear*; program(s) used to solve structure: *SHELXS97* (Sheldrick, 2008 ▶); program(s) used to refine structure: *SHELXL97* (Sheldrick, 2008 ▶); molecular graphics: *SHELXTL* (Sheldrick, 2008 ▶); software used to prepare material for publication: *SHELXTL*.

## Supplementary Material

Crystal structure: contains datablocks global, I. DOI: 10.1107/S160053680804395X/hb2886sup1.cif

Structure factors: contains datablocks I. DOI: 10.1107/S160053680804395X/hb2886Isup2.hkl

Additional supplementary materials:  crystallographic information; 3D view; checkCIF report

## Figures and Tables

**Table 1:** Hydrogen-bond geometry (Å, °)

*D*—H⋯*A*	*D*—H	H⋯*A*	*D*⋯*A*	*D*—H⋯*A*
N1—H1*A*⋯O2	0.905 (16)	1.925 (17)	2.653 (3)	136.3 (14)

**Fig. 1.**
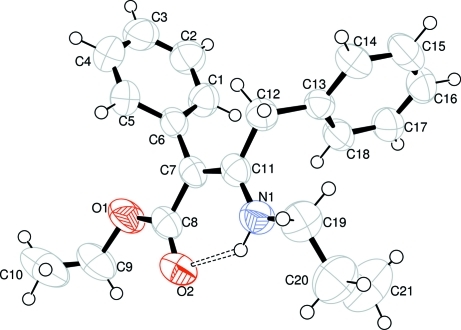
The molecular structure of (I) with 50% probability displacement ellipsoids. The dashed line indicates the intramolecular N—H···O hydrogen bond.

## supplementary crystallographic information

### Comment

Enamine compounds have been considered to be potential antibacterial agents (Xue *et al.*, 2007) and found important application in the synthesis of N-containint heterocylcles (Du *et al.*, 2006). To further study the structure and activity relationship, we determine the crystal structure of the title compound, (I).

In the molecular structure (Fig. 1), the torsion angles of N1—C11—C7—C6 and C8—C7—C11—C12 are -177.32 (17) and -175.14 (16)°, respectively. Furthermore, the distances C7—C11 and C11—N1 are 1.381 (3), 1.348 (3)Å, respectively.  Both of these features confirm the enamine structure formation. The two phenyl rings constructed an angle of 76.04 (12)°. The molecule adopts a *Z*-conformation, being stabilised by an intramolecular N—H···O hydrogen bond (Table 1).

### Experimental

The title compound was prepared according to the method of the literature (Du, *et al.*, 2006).  Colourless blocks of (I) were grown from a mixture of ethyl actate and petroleum ether.

### Refinement

The non-N H atoms were positioned geometrically (C—H = 0.93–0.98 Å) and refined as riding with *U*_iso_(H) = 1.2*U*_eq_(CH and CH_2_) or 1.5*U*_eq_(CH_3_). The N—H distance was refined with the restraint of 0.90 (1) Å, and the C19—C20, C20—C21, C9—C10 and C9—C10' with the restraint of 1.54 (1) Å. The ethyl radical of the ester moiety was found to be disordered, with the site occupancy ratio of 0.40 (2):0.60 (2).

### Crystal data

**Table:**

C_21_H_25_NO_2_	*F*(000) = 696
*M**_r_* = 323.42	*D*_x_ = 1.136 Mg m^−^^3^
Monoclinic, *P*2_1_/*n*	Mo *K*α radiation, λ = 0.71073 Å
Hall symbol: -P 2yn	Cell parameters from 4099 reflections
*a* = 12.186 (2) Å	θ = 2.2–27.1°
*b* = 8.4771 (17) Å	µ = 0.07 mm^−^^1^
*c* = 19.080 (4) Å	*T* = 293 K
β = 106.33 (3)°	Block, colourless
*V* = 1891.4 (7) Å^3^	0.28 × 0.22 × 0.18 mm
*Z* = 4	

### Data collection

**Table:**

Rigaku Saturn diffractometer	3323 independent reflections
Radiation source: rotating anode	2290 reflections with *I* > 2σ(*I*)
confocal	*R*_int_ = 0.034
Detector resolution: 7.31 pixels mm^-1^	θ_max_ = 25.0°, θ_min_ = 2.2°
ω scans	*h* = −14→14
Absorption correction: multi-scan (*CrystalClear*; Rigaku/MSC, 2005)	*k* = −9→10
*T*_min_ = 0.980, *T*_max_ = 0.987	*l* = −21→22
12325 measured reflections	

### Refinement

**Table:**

Refinement on *F*^2^	Secondary atom site location: difference Fourier map
Least-squares matrix: full	Hydrogen site location: inferred from neighbouring sites
*R*[*F*^2^ > 2σ(*F*^2^)] = 0.060	H atoms treated by a mixture of independent and constrained refinement
*wR*(*F*^2^) = 0.198	*w* = 1/[σ^2^(*F*_o_^2^) + (0.1207*P*)^2^ + 0.019*P*] where *P* = (*F*_o_^2^ + 2*F*_c_^2^)/3
*S* = 1.06	(Δ/σ)_max_ = 0.001
3323 reflections	Δρ_max_ = 0.24 e Å^−^^3^
232 parameters	Δρ_min_ = −0.19 e Å^−^^3^
5 restraints	Extinction correction: *SHELXL97* (Sheldrick, 2008), Fc^*^=kFc[1+0.001xFc^2^λ^3^/sin(2θ)]^-1/4^
Primary atom site location: structure-invariant direct methods	Extinction coefficient: 0.079 (13)

### Special details

**Table:**

Geometry. All e.s.d.'s (except the e.s.d. in the dihedral angle between two l.s. planes) are estimated using the full covariance matrix. The cell e.s.d.'s are taken into account individually in the estimation of e.s.d.'s in distances, angles and torsion angles; correlations between e.s.d.'s in cell parameters are only used when they are defined by crystal symmetry. An approximate (isotropic) treatment of cell e.s.d.'s is used for estimating e.s.d.'s involving l.s. planes.
Refinement. Refinement of *F*^2^ against ALL reflections. The weighted *R*-factor *wR* and goodness of fit *S* are based on *F*^2^, conventional *R*-factors *R* are based on *F*, with *F* set to zero for negative *F*^2^. The threshold expression of *F*^2^ > σ(*F*^2^) is used only for calculating *R*-factors(gt) *etc*. and is not relevant to the choice of reflections for refinement. *R*-factors based on *F*^2^ are statistically about twice as large as those based on *F*, and *R*- factors based on ALL data will be even larger.

### Fractional atomic coordinates and isotropic or equivalent isotropic displacement parameters (Å^2^)

**Table:**

	*x*	*y*	*z*	*U*_iso_*/*U*_eq_	Occ. (<1)
O1	0.98880 (16)	0.7415 (2)	0.24987 (10)	0.0936 (6)	
O2	1.07264 (14)	0.97745 (19)	0.24972 (8)	0.0791 (5)	
N1	0.95170 (17)	1.1941 (2)	0.16036 (11)	0.0710 (6)	
C1	0.7770 (2)	0.7362 (3)	0.07667 (13)	0.0723 (7)	
H1	0.8213	0.7655	0.0463	0.087*	
C2	0.6935 (2)	0.6239 (3)	0.05278 (16)	0.0864 (8)	
H2A	0.6812	0.5794	0.0067	0.104*	
C3	0.6286 (2)	0.5778 (3)	0.09719 (18)	0.0906 (8)	
H3A	0.5719	0.5018	0.0814	0.109*	
C4	0.6473 (2)	0.6434 (4)	0.16427 (18)	0.0977 (9)	
H4A	0.6038	0.6115	0.1947	0.117*	
C5	0.7302 (2)	0.7568 (3)	0.18764 (14)	0.0829 (8)	
H5A	0.7415	0.8007	0.2338	0.099*	
C6	0.79728 (17)	0.8072 (2)	0.14437 (11)	0.0591 (6)	
C7	0.88662 (17)	0.9301 (2)	0.16911 (10)	0.0572 (6)	
C8	0.99038 (19)	0.8903 (3)	0.22513 (12)	0.0654 (6)	
C9	1.0852 (3)	0.6899 (4)	0.30878 (18)	0.1159 (11)	0.60 (2)
H9A	1.1075	0.5855	0.2971	0.139*	0.60 (2)
H9B	1.1489	0.7602	0.3112	0.139*	0.60 (2)
C10	1.0665 (11)	0.684 (2)	0.3778 (4)	0.133 (4)	0.60 (2)
H10A	1.1353	0.6512	0.4133	0.199*	0.60 (2)
H10B	1.0063	0.6106	0.3770	0.199*	0.60 (2)
H10C	1.0450	0.7871	0.3904	0.199*	0.60 (2)
C9'	1.0852 (3)	0.6899 (4)	0.30878 (18)	0.1159 (11)	0.40 (2)
H9'A	1.1328	0.7787	0.3306	0.139*	0.40 (2)
H9'B	1.1314	0.6146	0.2913	0.139*	0.40 (2)
C10'	1.0339 (12)	0.616 (2)	0.3618 (8)	0.111 (4)	0.40 (2)
H10D	1.0935	0.5788	0.4030	0.166*	0.40 (2)
H10E	0.9871	0.5285	0.3392	0.166*	0.40 (2)
H10F	0.9879	0.6918	0.3779	0.166*	0.40 (2)
C11	0.87065 (17)	1.0815 (2)	0.14123 (11)	0.0588 (6)	
C12	0.75990 (17)	1.1280 (2)	0.08755 (11)	0.0618 (6)	
H12A	0.7336	1.2250	0.1045	0.074*	
H12B	0.7036	1.0471	0.0876	0.074*	
C13	0.76341 (16)	1.1525 (2)	0.00975 (11)	0.0565 (5)	
C14	0.6757 (2)	1.2334 (3)	−0.03842 (14)	0.0745 (7)	
H14A	0.6150	1.2716	−0.0228	0.089*	
C15	0.6773 (3)	1.2584 (3)	−0.11014 (15)	0.0918 (8)	
H15A	0.6185	1.3148	−0.1419	0.110*	
C16	0.7651 (2)	1.2003 (3)	−0.13433 (14)	0.0849 (8)	
H16A	0.7663	1.2175	−0.1822	0.102*	
C17	0.8495 (2)	1.1182 (3)	−0.08805 (14)	0.0803 (7)	
H17A	0.9085	1.0766	−0.1045	0.096*	
C18	0.84946 (18)	1.0953 (3)	−0.01624 (12)	0.0706 (6)	
H18A	0.9093	1.0397	0.0151	0.085*	
C19	0.9397 (2)	1.3609 (3)	0.14108 (16)	0.0837 (8)	
H19A	0.9085	1.4167	0.1755	0.100*	
H19B	0.8867	1.3728	0.0928	0.100*	
C20	1.0546 (3)	1.4324 (3)	0.14196 (19)	0.1120 (11)	
H20A	1.0446	1.5452	0.1344	0.134*	
H20B	1.1070	1.4167	0.1902	0.134*	
C21	1.1081 (3)	1.3697 (4)	0.0878 (3)	0.1381 (14)	
H21A	1.1817	1.4177	0.0946	0.207*	
H21B	1.0605	1.3929	0.0396	0.207*	
H21C	1.1169	1.2576	0.0937	0.207*	
H1A	1.0180 (11)	1.163 (2)	0.1924 (9)	0.065 (6)*	

### Atomic displacement parameters (Å^2^)

**Table:**

	*U*^11^	*U*^22^	*U*^33^	*U*^12^	*U*^13^	*U*^23^
O1	0.0861 (12)	0.0898 (12)	0.0851 (13)	0.0094 (9)	−0.0083 (10)	0.0275 (9)
O2	0.0732 (11)	0.0914 (11)	0.0625 (10)	0.0011 (8)	0.0026 (8)	−0.0069 (8)
N1	0.0710 (13)	0.0671 (11)	0.0688 (13)	−0.0032 (9)	0.0099 (11)	0.0003 (9)
C1	0.0817 (16)	0.0716 (13)	0.0667 (15)	−0.0068 (11)	0.0257 (13)	−0.0040 (11)
C2	0.0884 (17)	0.0779 (15)	0.0873 (18)	−0.0122 (13)	0.0156 (15)	−0.0051 (13)
C3	0.0755 (16)	0.0790 (16)	0.107 (2)	−0.0092 (12)	0.0090 (16)	0.0195 (15)
C4	0.0823 (18)	0.119 (2)	0.094 (2)	−0.0134 (16)	0.0297 (17)	0.0302 (18)
C5	0.0798 (17)	0.1103 (19)	0.0628 (15)	−0.0056 (14)	0.0270 (14)	0.0115 (13)
C6	0.0591 (12)	0.0645 (12)	0.0522 (12)	0.0079 (9)	0.0132 (10)	0.0094 (10)
C7	0.0629 (12)	0.0659 (12)	0.0428 (11)	0.0084 (9)	0.0148 (9)	0.0027 (9)
C8	0.0712 (14)	0.0745 (13)	0.0487 (12)	0.0090 (11)	0.0138 (11)	−0.0020 (11)
C9	0.096 (2)	0.133 (2)	0.094 (2)	0.0225 (18)	−0.0132 (18)	0.040 (2)
C10	0.102 (6)	0.194 (10)	0.081 (5)	0.043 (6)	−0.010 (4)	−0.022 (5)
C9'	0.096 (2)	0.133 (2)	0.094 (2)	0.0225 (18)	−0.0132 (18)	0.040 (2)
C10'	0.119 (8)	0.129 (9)	0.060 (6)	−0.016 (7)	−0.015 (5)	0.041 (6)
C11	0.0645 (12)	0.0669 (12)	0.0475 (11)	0.0055 (9)	0.0197 (10)	−0.0032 (9)
C12	0.0585 (12)	0.0668 (12)	0.0617 (13)	0.0101 (9)	0.0196 (10)	0.0044 (10)
C13	0.0568 (12)	0.0509 (10)	0.0576 (12)	−0.0005 (8)	0.0092 (10)	0.0023 (9)
C14	0.0717 (15)	0.0751 (14)	0.0690 (16)	0.0136 (11)	0.0071 (12)	0.0039 (12)
C15	0.095 (2)	0.0952 (18)	0.0692 (17)	0.0136 (14)	−0.0033 (15)	0.0173 (14)
C16	0.0953 (19)	0.0984 (18)	0.0556 (14)	−0.0162 (15)	0.0123 (14)	0.0129 (13)
C17	0.0747 (16)	0.1049 (18)	0.0626 (15)	0.0008 (13)	0.0213 (13)	0.0083 (13)
C18	0.0618 (13)	0.0874 (15)	0.0608 (14)	0.0117 (11)	0.0144 (11)	0.0121 (11)
C19	0.0904 (17)	0.0636 (14)	0.099 (2)	−0.0011 (12)	0.0290 (15)	−0.0085 (13)
C20	0.129 (3)	0.0726 (16)	0.138 (3)	−0.0143 (16)	0.044 (2)	−0.0030 (17)
C21	0.136 (3)	0.100 (2)	0.205 (4)	−0.006 (2)	0.092 (3)	−0.004 (2)

### Geometric parameters (Å, °)

**Table:**

O1—C8	1.349 (3)	C10'—H10E	0.9600
O1—C9	1.446 (3)	C10'—H10F	0.9600
O2—C8	1.227 (3)	C11—C12	1.499 (3)
N1—C11	1.348 (3)	C12—C13	1.511 (3)
N1—C19	1.458 (3)	C12—H12A	0.9700
N1—H1A	0.905 (10)	C12—H12B	0.9700
C1—C2	1.374 (3)	C13—C18	1.369 (3)
C1—C6	1.383 (3)	C13—C14	1.381 (3)
C1—H1	0.9300	C14—C15	1.390 (4)
C2—C3	1.369 (4)	C14—H14A	0.9300
C2—H2A	0.9300	C15—C16	1.370 (4)
C3—C4	1.355 (4)	C15—H15A	0.9300
C3—H3A	0.9300	C16—C17	1.346 (3)
C4—C5	1.375 (4)	C16—H16A	0.9300
C4—H4A	0.9300	C17—C18	1.384 (3)
C5—C6	1.383 (3)	C17—H17A	0.9300
C5—H5A	0.9300	C18—H18A	0.9300
C6—C7	1.485 (3)	C19—C20	1.522 (3)
C7—C11	1.381 (3)	C19—H19A	0.9700
C7—C8	1.448 (3)	C19—H19B	0.9700
C9—C10	1.399 (7)	C20—C21	1.467 (4)
C9—H9A	0.9700	C20—H20A	0.9700
C9—H9B	0.9700	C20—H20B	0.9700
C10—H10A	0.9600	C21—H21A	0.9600
C10—H10B	0.9600	C21—H21B	0.9600
C10—H10C	0.9600	C21—H21C	0.9600
C10'—H10D	0.9600		
			
C8—O1—C9	117.8 (2)	N1—C11—C12	116.66 (18)
C11—N1—C19	127.2 (2)	C7—C11—C12	120.63 (19)
C11—N1—H1A	114.9 (13)	C11—C12—C13	116.00 (16)
C19—N1—H1A	117.5 (13)	C11—C12—H12A	108.3
C2—C1—C6	122.2 (2)	C13—C12—H12A	108.3
C2—C1—H1	118.9	C11—C12—H12B	108.3
C6—C1—H1	118.9	C13—C12—H12B	108.3
C3—C2—C1	119.7 (3)	H12A—C12—H12B	107.4
C3—C2—H2A	120.2	C18—C13—C14	117.6 (2)
C1—C2—H2A	120.2	C18—C13—C12	122.92 (19)
C4—C3—C2	119.6 (3)	C14—C13—C12	119.44 (18)
C4—C3—H3A	120.2	C13—C14—C15	120.5 (2)
C2—C3—H3A	120.2	C13—C14—H14A	119.7
C3—C4—C5	120.4 (2)	C15—C14—H14A	119.7
C3—C4—H4A	119.8	C16—C15—C14	120.4 (2)
C5—C4—H4A	119.8	C16—C15—H15A	119.8
C4—C5—C6	121.8 (2)	C14—C15—H15A	119.8
C4—C5—H5A	119.1	C17—C16—C15	119.3 (2)
C6—C5—H5A	119.1	C17—C16—H16A	120.3
C1—C6—C5	116.3 (2)	C15—C16—H16A	120.3
C1—C6—C7	121.66 (17)	C16—C17—C18	120.6 (2)
C5—C6—C7	122.1 (2)	C16—C17—H17A	119.7
C11—C7—C8	120.0 (2)	C18—C17—H17A	119.7
C11—C7—C6	121.21 (19)	C13—C18—C17	121.5 (2)
C8—C7—C6	118.74 (18)	C13—C18—H18A	119.2
O2—C8—O1	121.3 (2)	C17—C18—H18A	119.2
O2—C8—C7	126.3 (2)	N1—C19—C20	110.9 (2)
O1—C8—C7	112.4 (2)	N1—C19—H19A	109.5
C10—C9—O1	115.5 (5)	C20—C19—H19A	109.5
C10—C9—H9A	108.4	N1—C19—H19B	109.5
O1—C9—H9A	108.4	C20—C19—H19B	109.5
C10—C9—H9B	108.4	H19A—C19—H19B	108.0
O1—C9—H9B	108.4	C21—C20—C19	115.9 (3)
H9A—C9—H9B	107.5	C21—C20—H20A	108.3
C9—C10—H10A	109.5	C19—C20—H20A	108.3
C9—C10—H10B	109.5	C21—C20—H20B	108.3
H10A—C10—H10B	109.5	C19—C20—H20B	108.3
C9—C10—H10C	109.5	H20A—C20—H20B	107.4
H10A—C10—H10C	109.5	C20—C21—H21A	109.5
H10B—C10—H10C	109.5	C20—C21—H21B	109.5
H10D—C10'—H10E	109.5	H21A—C21—H21B	109.5
H10D—C10'—H10F	109.5	C20—C21—H21C	109.5
H10E—C10'—H10F	109.5	H21A—C21—H21C	109.5
N1—C11—C7	122.7 (2)	H21B—C21—H21C	109.5
			
C6—C1—C2—C3	−0.9 (4)	C19—N1—C11—C12	7.4 (3)
C1—C2—C3—C4	0.0 (4)	C8—C7—C11—N1	4.6 (3)
C2—C3—C4—C5	0.6 (4)	C6—C7—C11—N1	−177.32 (17)
C3—C4—C5—C6	−0.3 (4)	C8—C7—C11—C12	−175.14 (16)
C2—C1—C6—C5	1.2 (3)	C6—C7—C11—C12	2.9 (3)
C2—C1—C6—C7	−179.1 (2)	N1—C11—C12—C13	71.9 (2)
C4—C5—C6—C1	−0.5 (4)	C7—C11—C12—C13	−108.3 (2)
C4—C5—C6—C7	179.7 (2)	C11—C12—C13—C18	17.9 (3)
C1—C6—C7—C11	74.3 (3)	C11—C12—C13—C14	−163.29 (19)
C5—C6—C7—C11	−105.9 (2)	C18—C13—C14—C15	−1.5 (3)
C1—C6—C7—C8	−107.6 (2)	C12—C13—C14—C15	179.6 (2)
C5—C6—C7—C8	72.1 (3)	C13—C14—C15—C16	1.2 (4)
C9—O1—C8—O2	2.7 (3)	C14—C15—C16—C17	0.3 (4)
C9—O1—C8—C7	−176.8 (2)	C15—C16—C17—C18	−1.4 (4)
C11—C7—C8—O2	−3.9 (3)	C14—C13—C18—C17	0.4 (3)
C6—C7—C8—O2	178.02 (18)	C12—C13—C18—C17	179.3 (2)
C11—C7—C8—O1	175.63 (17)	C16—C17—C18—C13	1.1 (4)
C6—C7—C8—O1	−2.5 (2)	C11—N1—C19—C20	−154.4 (2)
C8—O1—C9—C10	103.2 (10)	N1—C19—C20—C21	64.4 (4)
C19—N1—C11—C7	−172.39 (19)		

### Hydrogen-bond geometry (Å, °)

**Table:**

*D*—H···*A*	*D*—H	H···*A*	*D*···*A*	*D*—H···*A*
N1—H1A···O2	0.91 (2)	1.93 (2)	2.653 (3)	136 (1)
